# Longitudinal changes in anxiety and psychological distress, and associated risk and protective factors during the first three months of the COVID‐19 pandemic in Germany

**DOI:** 10.1002/brb3.1964

**Published:** 2020-11-23

**Authors:** Antonia Bendau, Jens Plag, Stefanie Kunas, Sarah Wyka, Andreas Ströhle, Moritz Bruno Petzold

**Affiliations:** ^1^ Charité ‐ Universitätsmedizin Berlin, corporate member of Freie Universität Berlin, Humboldt‐Universität zu Berlin, and Berlin Institute of Health ‐ Department for Psychiatry and Psychotherapy (CCM) Berlin Germany

**Keywords:** Corona, depression, mental disorder, mental health, Sars‐CoV‐2

## Abstract

**Background:**

The COVID‐19 pandemic is related to multiple stressors and therefore may be associated with psychological distress. The aim of this study was to longitudinally assess symptoms of (un‐)specific anxiety and depression along different stages of the pandemic to generate knowledge about the progress of psychological consequences of the pandemic and to test the role of potential risk and resilience factors that were derived from cross‐sectional studies and official recommendations.

**Methods:**

The present study uses a longitudinal observational design with four waves of online data collection (from March 27 to June 15, 2020) in a convenience sample of the general population in Germany. A total of *N* = 2376 participants that completed at least two waves of the survey were included in the analyses.

**Findings:**

Specific COVID‐19‐related anxiety and the average daily amount of preoccupation with the pandemic decreased continuously over the four waves. Unspecific worrying and depressive symptoms decreased on average but not on median level. Self‐efficacy, normalization, maintaining social contacts, and knowledge, where to get medical support, were associated with fewer symptoms relative to baseline. Suppression, unhealthy habits, and a longer average daily time of thinking about the pandemic were correlated with a relative increase of symptoms.

**Interpretation:**

Our findings provide insight into the longitudinal changes of symptoms of psychological distress along the first three months of the COVID‐19 pandemic in Germany. Furthermore, we were able to reaffirm the anticipated protective and risk factors that were extracted from previous studies and recommendations.

## Graphical abstract

Longitudinal assessments revealed that symptoms of anxiety and psychological distress related to the COVID‐19 pandemic decreased on average continuously across the first three months of the pandemic in Germany. Self‐efficacy, normalization, maintaining social contacts, and knowledge, where to get medical support, were longitudinally associated with better mental health outcomes. In contrast, suppression, unhealthy habits, and ruminating about the pandemic were correlated with a higher symptom severity.

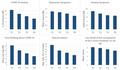

## 1 INTRODUCTION

Since the first cases of pneumonia of unknown cause were detected in Wuhan, China, in December 2019, the new SARS‐CoV‐2 virus has rapidly spread throughout the world, and this pandemic represents one of the most severe international health problems in the last decades (Ghebreyesus, 2020; Zhu et al., 2020). The disease itself, as well as the measures to fight the pandemic, may have the potential to cause psychological distress in large parts of the populations worldwide (Helmy et al., 2020; Torales et al., 2020; Xiang et al., 2020). This gives research regarding the mental health consequences of the pandemic a high priority (O'Connor et al., 2020). Although enormous efforts by the research community which led to a high number of publications of heterogeneous quality (Rzymski et al., 2020), substantial research on the psychosocial consequences of the pandemic is still missing.

First cross‐sectional studies from convenience samples, for example, in China, Italy, Spain, or the United States show that substantial parts of the participants report symptoms of depression, anxiety, and distress as a result of the pandemic (Fitzpatrick et al., 2020; González‐Sanguino et al., 2020; Mazza et al., 2020; Qiu et al., 2020; Wang et al., 2020a). These studies can be interpreted rather as first hints that psychological consequences of the pandemic might occur then as robust evidence due to several methodological shortcomings, for example, missing baseline assessments prior to the pandemic. More robust evidence comes from a study with a representative sample from the general population of the United States with over 300.000 participants. One in three participants screened positive for depression and/or anxiety disorders, and participants were more than three times as likely to show positive screening for depression and/or anxiety disorders compared to a baseline sample in 2019 (Twenge & Joiner, 2020). Regarding the situation in Germany, three cross‐sectional studies showed elevated levels of depression and anxiety and were able to identify several risk factors (e.g., high level of media consumption, higher substance use, and suppression of negative emotions) and protective factors (e.g., regular physical activity, higher self‐efficacy, maintaining social contacts, and trust in government actions) (Bäuerle et al., 2020; Bendau et al., 2020; Petzold, Bendau, Plag, Pyrkosch, Mascarell Maricic, et al., 2020). These factors are similar to those in the context of, for example, Ebola (D’Agostino et al., 2017) and Zika outbreaks (Dillard et al., 2018).

Although these cross‐sectional studies delivered timely and important first insight into the mental health consequences of the pandemic, they come with several shortcomings. First, the COVID‐19 pandemic is a highly dynamic situation where mental health consequences might change rapidly due to, for example, changing case numbers, changing governmental restrictions, habituation or change in media coverage. Therefore, longitudinal research with ideally periodically repeated measurements is needed to give insights into the progress of psychological consequences of the pandemic and their longitudinal associations with risk and protective factors. To our knowledge, there is only one study with two points of measurements regarding the mental health consequences of the COVID‐19 pandemic: Wang et al. (2020b) followed 333 participants from the Chinese general population, which participated in an online survey in January and March 2020. The study does only present cross‐sectional associations at the two points of measurement and not longitudinal associations of risk and resilience factors. With regard to findings from previous SARS outbreaks (Bell & Wade, 2020; Leung et al., 2005) and the H1N1 influenza (“swine flu”) pandemic (Bults et al., 2011), an expected pattern of the change of symptoms across time can be derived: The majority of the surveyed individuals expressed high amounts of anxiety at the initial phase of the outbreaks which subsequently decreased across the further progress of the epi‐/pandemic.

To contribute to the prevention of mental health consequences of the COVID‐19 pandemic, several international organizations published first recommendations (IASC, 2020; IFRC, 2020; WHO, 2020). These recommendations focus mainly on general knowledge of pandemics, traumatic events, and resilience research. In the very dynamic situation of the COVID‐19 pandemic, substantial research on its mental health consequences and potential risk and resilience factors that put the existing recommendations on a stronger empirical basis seems to be of extraordinary importance (Horesh & Brown, 2020; Torales et al., 2020).

This study aimed to describe the psychological consequences of the pandemic in the general population in Germany in a longitudinal design to generate knowledge on the progress of symptoms and on factors that are associated with later mental distress. Testing the role of potential risk and resilience factors that were derived from cross‐sectional studies might lay the basis for recommendations regarding the protection of the mental health in the pandemic.

## 2 METHODS

### 2.1 Design

The present study uses a longitudinal observational design with four waves of data collection in a convenience sample of the general population in Germany (Petzold, Bendau, Plag, Pyrkosch, Mascarell Maricic, et al., 2020). Prior to recruitment, the study was approved by the ethics committee of Charité‐Universitätsmedizin Berlin (EA1/071/20) and registered on clinicaltrials.gov (NCT04331106).

### 2.2 Recruitment

A longitudinal online survey via SoSci Survey was used to examine the changes in depressive and anxiety symptoms during the first three months of the pandemic. Primarily the official social media channels (Twitter, Facebook, and Instagram) and the website of the Charité‐Universitätsmedizin Berlin and a few news portals were used for recruitment of the first wave of data collection. An invitation to participate in the study with the attached link to the survey was posted on each channel once. We did not use paid advertising, and no compensation was offered. Individuals who entered their e‐mail‐addresses and gave their consent were contacted for the next waves via e‐mail. Data were stored separately from contact information and merged via anonymous codes. Only individuals that participated in at least two waves of data collection were included in the analyses (*N* = 2376; see Figure S1). *N* = 1070 completed two waves, *N* = 803 three waves, and *N* = 503 all four waves. Prior to participation, all participants gave informed consent.

**Figure 1:**
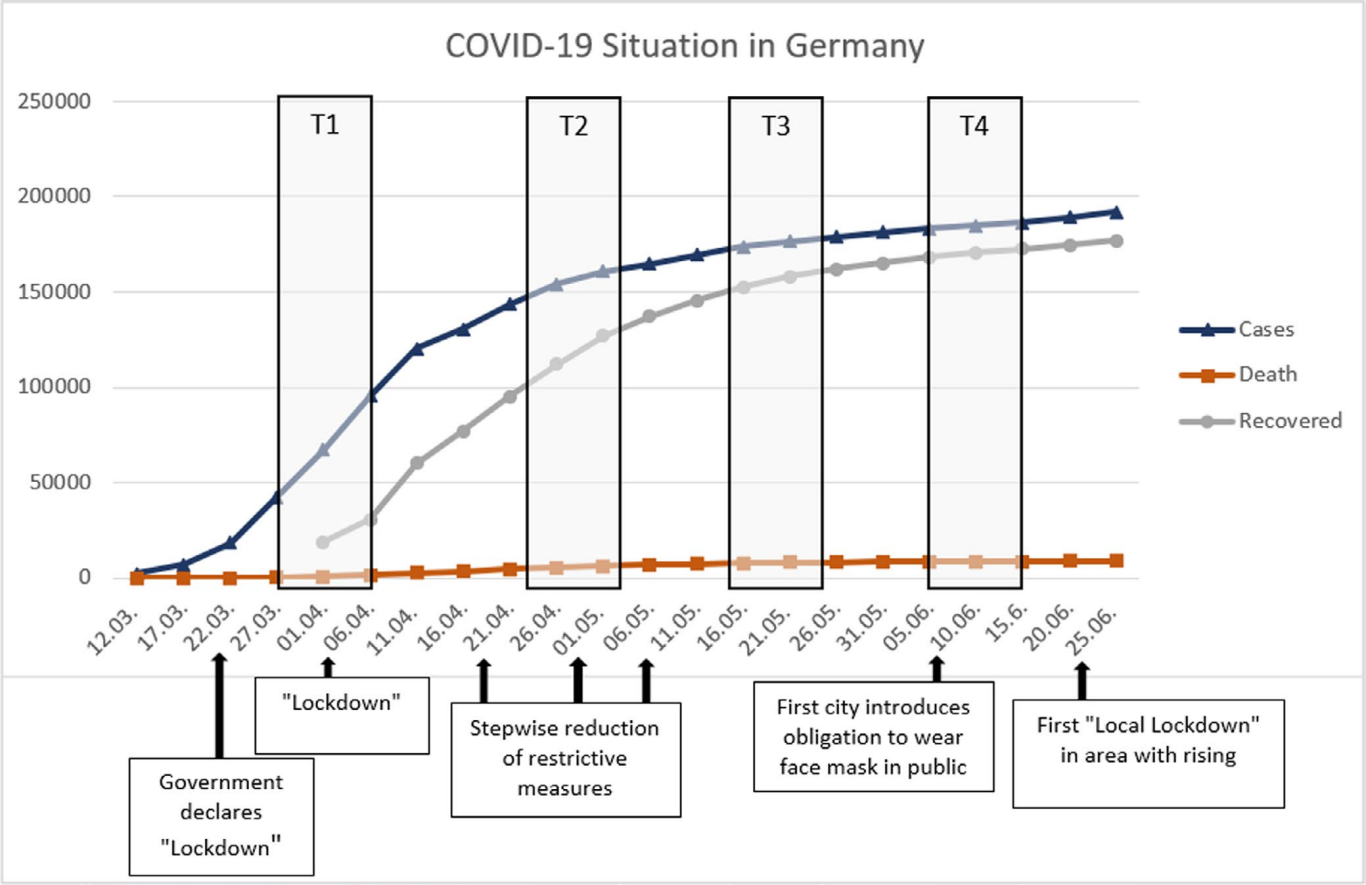
COVID‐19 Situation during recruitment: cases, deaths, recovered and political measures

The first period of data collection (T1) took part from March 27 to April 6, 2020. The second assessment (T2) started on April 24 and ended on May 4. The third period (T3) lasted from May 15 to May 25 and the fourth (T4) from June 6 to June 15. Figure 1 illustrates the situation in Germany regarding COVID‐19 during the four periods of data collection in terms of infected cases, deaths, recoveries, and political measures.

During the first data collection, the number of infected individuals grew fast (see Figure 1) and shortly before, strict restrictions became effective nationwide to reduce the infection rates (e.g., physical distancing and closure of most institutions and shops) (Mitteldeutscher Rundfunk, 2020; Robert Koch Institut, 2020). Alongside the second period, the growth of infections was decreased, and first alleviations of the preventive measures appeared, but most restrictions were the same as during the first period. During the third and the fourth periods, there were very slowly rising infection numbers and many preventive measures removed.

### 2.3 Eligibility criteria

To be able to participate, the minimum age of 18 years, the current residence in Germany, and the ability to complete the questionnaires in German were required. Other inclusion or exclusion criteria did not apply.

### 2.4 Assessment

The same questionnaire was used for all four assessments and required approximately 10 to 15 minutes to be completed. Solely demographics were only collected at the first wave. The personal confrontation with the virus (e.g., being in quarantine, being tested or diagnosed for COVID‐19) was surveyed at all measurement periods as well as the preoccupation with the pandemic (e.g., daily amount of thinking about the pandemic and the subjective risk of getting infected).

To analyze specific anxiety symptoms related to the COVID‐19 pandemic, the COVID‐19‐Anxiety Questionnaire (C‐19‐A; Petzold, Bendau, Plag, Pyrkosch, Maricic, et al., 2020) was used. This self‐report scale consists of ten items, which occurrence is rated on a 5‐point Likert scale from 0 (“never”) to 4 (“all the time”). Moreover, different aspects of fears regarding the pandemic were recorded with nine items on a 6‐point Likert scale from 1 (“not true at all”) to 6 (“totally true”) (Petzold, Bendau, Plag, Pyrkosch, Maricic, et al., 2020). The validated Patient Health Questionnaire‐4 (PHQ‐4) (Löwe et al., 2010) was used to assess psychological distress, respectively, to screen for unspecific anxiety (GAD‐2 subscale, two items) and depressive symptoms (PHQ‐2 subscale, two items). The intensity of the items is rated on a 4‐point Likert scale from 0 (“not at all”) to 3 (“nearly every day”). A sum score of 3 on the subscales, respectively, 6 on the total score remarks the cutoff for a substantial symptom severity.

Moreover, eight items targeting potential protective factors in dealing with the pandemic (e.g., self‐efficacy and acceptance) and five items concerning potential risk factors (e.g., suppression and substance use) were included in the survey. The items were derived from the recommendations of the IASC (2020). All items were rated on a 6‐point Likert scale, ranging from 1 (“not true at all”) to 6 (“totally true”).

### 2.5 Analyses

SPSS Statistics Version 25 was used for all analyses, and the significance level was set to .05 (two‐tailed). Missing data were handled by casewise‐deletion. Descriptive statistics, Pearson’s partial correlations (with partialization of the baseline T1‐values), and analyses of variance with post hoc analysis were used for data analysis. Not all variables were distributed normally but we applied those methods nevertheless because they are rather robust with respect to non‐normality (Norman, 2010). The correlations were computed for the changes within the short interval T1 to T2 (about four weeks) and the long interval T1 to T4 (about ten weeks) to examine the probably most different changes over time.

## 3 RESULTS

### 3.1 Sample characteristics

76.7 % of the included participants were female (*N* = 1822), 22.8 % male (*N* = 542), and 0.5 % reported to identify as diverse (*N* = 12). Mean age at T1 was 38.76 years (*SD* = 12.01, Range 18–82). 10.4 % of the sample had a secondary school degree or lower (*N* = 246), 24.5 % reported a higher education entrance qualification (*N* = 582), and 43.2 % reported a university degree (*N* = 1027). 393 participants reported to work in a medical context (16.5 %). Table S1 shows details on the sample characteristics at the different measurement waves.

### 3.2 Exposure with COVID‐19

**Figure 2:**
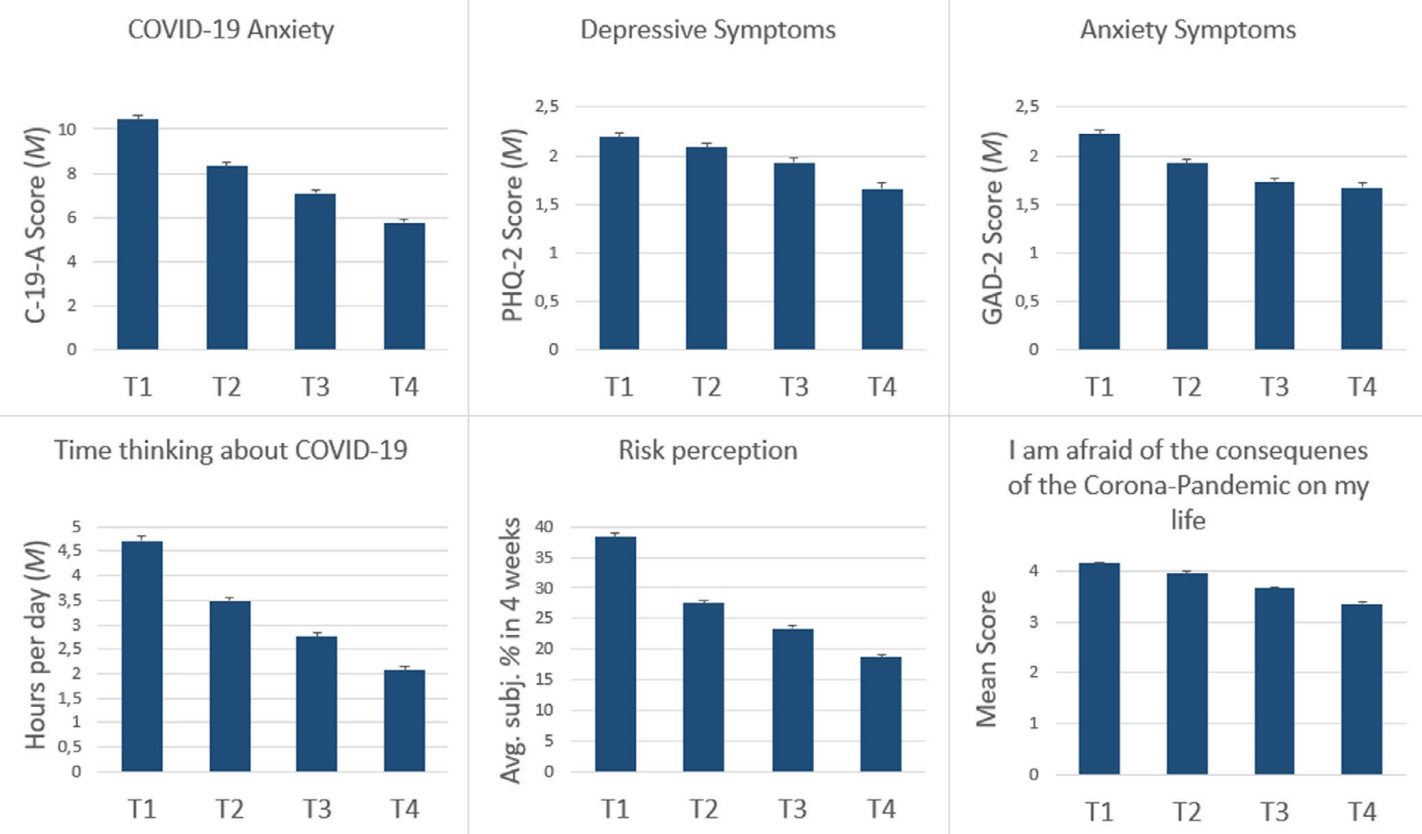
Development of Anxiety, Depression and COVID‐19 associated cognitions over time

The relative proportion of participants who knew people that had already been infected with COVID‐19 rose continuously from 26.5% at T1 via 37.7 % (T2) and 41.0 % (T3) to 41.6 % at T4. The proportion of those who suspected themselves to be infected (28.4–30.1–31.8–33.1 %) and those who had been tested for COVID‐19 (3.9–7.4–8.7–11.1 %) also increased slightly along the four assessment periods. In contrast, the percentage of individuals diagnosed with COVID‐19 remained at around 1%, and the relative number of individuals in quarantine decreased (5.6–2.3–1.7–0.9 %). In contrast to the still rising number of infected individuals, the average subjective risk of becoming infected with the virus within the next month decreased continuously from 38.3 % to 18.7 % (see Figure 2). Furthermore, the average daily amount of time spent thinking about COVID‐19 followed the same pattern (Figure 2): It has been more than halved from T1 (almost 5 hours) to T4 (2 hours).

### 3.3 Changes in symptoms of COVID‐19‐specific and unspecific anxiety and depression

Table 1 shows the changes in symptoms of COVID‐19‐specific fears (C‐19‐A), unspecific anxiety, and depression (GAD‐2, PHQ‐2, and PHQ‐4) over the four measurement periods. There was a clear and significant continuous downward trend of all four scales visible at the mean value level (see Figure 2). This was driven mainly by people who show a particularly strong reduction in symptom severity (see negative values of the 10. and 25. percentiles of the differences in Table 1). While the median, as well as the most frequent value, was negative for changes in specific COVID‐19 anxiety, the median and the modus were zero (indicates no change) for the PHQ‐4 and its subscales (except the median of the T4‐T1 difference).

**Table 1:** Changes in symptoms of COVID‐19‐specific and unspecific anxiety and depression

		COVID‐19‐related anxiety (C‐19‐A)	Depressive and anxiety symptoms (PHQ‐4)	Depressive symptoms (PHQ‐2)	Unspecific Anxiety symptoms (GAD‐2)
T1 (*N* = 1855)	Median	9.00	4.00	2.00	2.00
	> cutoff (%)		31.00	32.70	36.40
T2 (*N* = 1804)	Median	7.00	3.00	2.00	2.00
	> cutoff (%)		25.90	30.50	29.20
T3 (*N* = 1512)	Median	5.00	3.00	2.00	1.00
	> cutoff (%)		22.10	25.20	24.50
T4 (*N* = 1328)	*M* ± SD	4.00	3.00	2.00	1.00
	Median > cutoff (%)		22.60	25.30	24.90
Difference T2 – T1 (*N* = 1336)	*M* ± SD	−2.02 ± 4.89	−0.30 ± 2.26	−0.04 ± 1.35	−0.26 ± 1.35
	Modus	−1.00	0.00	0.00	0.00
	Median	−2.00	0.00	0.00	0.00
	10. Percentile	−8.00	−3.00	−2.00	−2.00
	25. Percentile	−5.00	−2.00	−1.00	−1.00
	75. Percentile	1.00	1.00	1.00	0.00
	90. Percentile	4.00	2.00	2.00	1.00
Effect size	Cohen’s d (p)	0.41 (<.001^***^)	0.13 (<.001^***^)	0.03 (.230)	0.19 (<.001^***^)
Difference T4 – T1 (*N* = 964)	*M* ± SD	−4.50 ± 5.49	−0.77 ± 2.65	−0.27 ± 1.53	−0.49 ± 1.50
	Modus	−2.00	0.00	0.00	0.00
	Median	−4.00	−1.00	0.00	0.00
	10. Percentile	−11.00	−4.00	−2.00	−2.00
	25. Percentile	−8.00	−2.00	−1.00	−1.00
	75. Percentile	−1.00	1.00	0.00	0.00
	90. Percentile	1.00	2.00	2.00	1.00
Effect size	Cohen’s d (p)	0.82 (<.001^***^)	0.29 (<.001^***^)	0.18 (<.001^***^)	0.33 (<.001^***^)

Significance of the differences (*p*) was computed with Bonferroni adjusted paired t‐tests and the effect size with Cohen’s *d*. *significant at .05‐level, **significant at .01‐level, ***significant at .001‐level.

Table 2 shows the progress of different aspects of fears related to the COVID‐19 pandemic over time. While the fear of becoming infected, the fear of the consequences of the pandemic in general, and the fear of economic consequences showed continuous downward trends, the fear of health‐related consequences stayed on average level somewhat stable. The fear of social consequences increased slightly from T1 to T2 and decreased to T3 and T4.

**Table 2:** Changes in different aspects of COVID‐19‐related fears

… I am afraid …		… to get infected with Corona	… of the consequences of the Corona Pandemic on my life	… of the consequences for my health if I get infected	… of the social consequences of Corona	… of the economic consequences of Corona on my life
T1 (*N* = 1855)	*M* ± SD	3.39 ± 1.50	4.14 ± 1.46	3.56 ± 1.61	3.84 ± 1.6	3.23 ± 1.73
	Median	3.00	4.00	3.00	4.00	3.00
T2 (*N* = 1808)	*M* ± SD	3.20 ± 1.50	3.95 ± 1.43	3.60 ± 1.61	3.99 ± 1.57	3.06 ± 1.66
	Median	3.00	4.00	4.00	4.00	3.00
T3 (*N* = 1537)	*M* ± SD	3.13 ± 1.48	3.65 ± 1.49	3.59 ± 1.61	3.61 ± 1.60	2.83 ± 1.62
	Median	3.00	4.00	4.00	4.00	2.00
T4 (*N* = 1345)	*M* ± SD	3.07 ± 1.52	3.35 ± 1.46	3.58 ± 1.62	3.37 ± 1.56	2.68 ± 1.59
	Median	3.00	3.00	4.00	3.00	2.00
Difference T2 – T1 (*N* = 1340)	*M* ± SD	−0.15 ± 1.18	−0.20 ± 1.33	0.11 ± 1.28	0.12 ± 1.51	−0.19 ± 1.42
	Median	0.00	0.00	0.00	0.00	0.00
	10. Percentile	−2.00	−2.00	−1.00	−2.00	−2.00
	25. Percentile	−1.00	−1.00	−1.00	−1.00	−1.00
	75. Percentile	1.00	1.00	1.00	1.00	0.00
	90. Percentile	1.00	1.00	2.00	2.00	1.00
Difference T4 – T1 (*N* = 975)	*M* ± SD	−0.30 ± 1.30	−0.78 ± 1.38	−0.05 ± 1.41	−0.43 ± 1.50	−0.51 ± 1.45
	Median	0.00	−1.00	0.00	0.00	0.00
	10. Percentile	−2.00	−2.00	−2.00	−2.00	−2.00
	25. Percentile	−1.00	−1.00	‐1.00	‐1.00	‐1.00
	75. Percentile	0.00	1.00	1.00	0.00	0.00
	90. Percentile	1.00	1.00	2.00	2.00	1.00

Furthermore, also the rating of the own anxiety regarding COVID‐19 as being exaggerated as well as the rating of the statement that this anxiety leads to limitations in daily life followed a continuous downward trend over time on average level (see Table S2).

### 3.4 Risk and protective factors

Table 3 shows the partial correlations of COVID‐19‐specific fears and symptoms of unspecific anxiety and depression (at T2 and T4) with potential protective and risk factors (at T1). In all partial correlations the baseline T1‐values of the C‐19‐A score, respectively, PHQ‐4, PHQ‐2, and GAD‐2, were partialized to assess the associations of the protective and risk factors with the relative changes over time and not with absolute values at single time points (see Petzold, Bendau, Plag, Pyrkosch, Mascarell Maricic, et al., 2020 for correlations with absolute values).

**Table 3:** Associations of COVID‐19‐specific fears and symptoms of unspecific anxiety and depression (at T2 and T4) with protective and risk factors (at T1). Pearson’s partial correlations with partialization of the T1‐values of the C‐19‐A, respectively, PHQ‐4, PHQ‐2, and GAD‐2)

	COVID‐19‐related anxiety (C‐19‐A) [T2] [T4]	Depressive & anxiety symptoms (PHQ‐4) [T2] [T4]	Depressive symptoms (PHQ‐2) [T2] [T4]	Unspecific Anxiety symptoms (GAD‐2) [T2] [T4]
Protective factors [T1]				
Self‐efficacy general	*r *= −.09 (*p *= .001***) *r *= −.10 (*p *= .002**)	*r *= −.07 (*p *= .013*) *r *= −.10 (*p *= .003**)	*r *= −.07 (*p *= .010**) *r *= −.10 (*p *= .003**)	*r *= −.12 (*p *< .001***) *r *= −.14 (*p *< .001***)
Self‐efficacy health	*r *= −.08 (*p *= .002**) *r *= −.21 (*p *< .001***)	*r *= .03 (*p *= .314) *r *= −.12 (*p *< .001***)	*r *= .03 (*p *= .247) *r *= −.13 (*p *< .001***)	*r *= .02 (*p *= .442) *r *= −.12 (*p *< .001***)
Self‐efficacy social	*r *= −.07 (*p *= .007**) *r *= −.07 (*p *= .026*)	*r *= −.11 (*p *< .001***) *r *= −.10 (*p *= .002**)	*r *= −.13 (*p *< .001***) *r *= −.10 (*p *= .002**)	*r *= −.13 (*p *< .001***) *r *= −.14 (*p *< .001***)
Self‐efficacy economic	*r *= −.04 (*p *= .196) *r *= −.04 (*p *= .250)	*r *= −.03 (*p *= .298) *r *= −.08 (*p *= .012*)	*r *= −.02 (*p *= .417) *r *= −.08 (*p *= .015*)	*r *= −.07 (*p *= .016*) *r *= −.10 (*p *= .002**)
Normalization	*r *= −.09 (*p *= .002**) *r *= −.05 (*p *= .131)	*r *= −.04 (*p *= .140) *r *= −.03 (*p *= .323)	*r *= −.05 (*p *= .074) *r *= −.07 (*p *= .038*)	*r *= −.08 (*p *= .006**) *r *= −.04 (*p *= .238)
Social contacts	*r *= −.05 (*p *= .105) *r *= −.00 (*p *= .960)	*r *= −.13 (*p *< .001***) *r *= −.04 (*p *= .212)	*r *= −.15 (*p *< .001***) *r *= −.05 (*p *= .109)	*r *= −.12 (*p *< .001***) *r *= −.06 (*p *= .047*)
Medical Support	*r *= −.05 (*p *= .099) *r *= −.10 (*p *= .002**)	*r *= −.06 (*p *= .023*) *r *= −.14 (*p *< .001***)	*r *= −.08 (*p *= .003**) *r *= −.13 (*p *< .001***)	*r *= −.06 (*p *= .030*) *r *= −.15 (*p *< .001***)
Psychological Support	*r *= −.02 (*p *= .438) *r *= −.09 (*p *= .004**)	*r *= −.01 (*p *= .770) *r *= −.02 (*p *= .620)	*r *= −.04 (*p *= .142) *r *= −.01 (*p *= .852)	*r *= .01 (*p *= .660) *r *= .02 (*p *= .456)
Risk factors [T1]				
Suppression	*r *= .06 (*p *= .019*) *r *= .03 (*p *= .317)	*r *= −.04 (*p *= .139) *r *= −.01 (*p *= .844)	*r *= .04 (*p *= .121) *r *= .03 (*p *= .338)	*r *= .09 (*p *= .001***) *r *= .02 (*p *= .637)
Reduced physical activity	*r *= .00 (*p *= .986) *r *= .06 (*p *= .055)	*r *= .01 (*p *= .837) *r *= .04 (*p *= .195)	*r *= .04 (*p *= .187) *r *= .04 (*p *= .217)	*r *= .00 (*p *= .959) *r *= .06 (*p *= .071)
Reduced healthy diet	*r *= .02 (*p *= .393) *r *= .08 (*p *= .019*)	*r *= .07 (*p *= .013*) *r *= .04 (*p *= .229)	*r *= .10 (*p *< .001***) *r *= .03 (*p *= .426)	*r *= .05 (*p *= .052) *r *= .08 (*p *= .019*)
More substance use	*r *= .08 (*p *= .003**) *r *= .11 (*p *= .001***)	*r *= .07 (*p *= .009**) *r *= .11 (*p *= .001***)	*r *= .05 (*p *= .082) *r *= .12 (*p *< .001***)	*r *= .11 (p < .001***) *r *= .11 (*p *= .001***)
h/day thinking about	*r *= .04 (*p *= .145) *r *= .08 (*p *= .016*)	*r *= .01 (*p *= .865) *r *= .02 (*p *= .497)	*r *= .03 (*p *= .399) *r *= .07 (*p *= .042*)	*r *= .02 (*p *= .413) *r *= .02 (*p *= .541)

^*^Significant at .05‐level, **significant at .01‐level, ***significant at .001‐level; bold values represent significant values of a size of at least .1.

General self‐efficacy and social self‐efficacy were significantly negatively correlated with all four scales of both examined follow‐up periods (T2 and T4). Health‐related self‐efficacy showed significantly negative correlations with T4 but not with T2 values (except regarding C‐19‐A with significant negative correlations at both intervals). The same applied for self‐efficacy regarding the economic consequences. In contrast, the fostering of social contacts showed significant negative correlations with unspecific anxiety and depressive symptoms especially in the short run (at T2) and rather not with the T4 values.

While the knowledge where to get medical treatment, if required, was significantly associated with fewer symptoms of (un‐)specific anxiety and depression, the knowledge where to get psychosocial treatment showed no significant correlations (except with C‐19‐A at T4). Normalization was associated with less anxiety‐related burden at T2 (C‐19‐A and GAD‐2) and less depressive symptoms at T4 (PHQ‐2).

Increased substance use was the strongest of the five risk factors. It was significantly associated with more psychological strain at both: the short (T2) and the long run (T4). Suppression, the daily amount of preoccupation with the COVID‐19 topic, and a reduced healthy diet showed only scattered significantly positive associations with (un‐)specific anxiety and depression.

Regarding changes in the rating of the own anxiety regarding COVID‐19 as being exaggerated as well as the rating of the statement that this anxiety leads to limitations in daily life, all five risk factors showed significant associations with more anxiety burden at both time perspectives in almost all variables (see Table S3).

Regarding the different aspects of COVID‐19‐related fears (see Table S4), general self‐efficacy was particularly associated with less fear regarding the consequences of the pandemic in general and social self‐efficacy was particularly associated negatively with fearing the social consequences. Following the same pattern, health‐related self‐efficacy showed the strongest correlation with a reduction of health‐related fears and economic self‐efficacy with less fearing the economic consequences of the pandemic.

## 4 DISCUSSION

In this study, we examined the changes over time in symptoms of COVID‐19‐related anxiety unspecific anxiety, and depression along the first three months of the COVID‐19 pandemic in Germany. Furthermore, we examined several risk and protective factors in this context.

First, it became evident that especially the COVID‐19‐specific fear (C‐19‐A) showed a consistent decrease over the four measurements. The same trend is evident regarding the daily amount of preoccupation with the topic of COVID‐19 and the subjective risk perception. This is in line with observations of psychological reactions to previous outbreaks of high‐risk infectious diseases (Bell & Wade, 2020; Bults et al., 2011; Leung et al., 2005) with a peak of symptoms of anxiety and psychological distress early during outbreaks and a subsequent decrease as time proceeds.

In contrast, unspecific anxiety and especially depressive symptoms showed a slighter decrease—with almost no changes on median level. Those results seem plausible because a more general worrying and depressive symptoms are broader constructs than the fear of a specific matter, may reflect suffering of, for example, social or economic consequences of the pandemic, and may have a stronger tendency to persist over time than symptoms of acute specific anxiety. This would be in line with results of Chong et al. (2004) from a SARS outbreak that indicate that depressive symptoms are the predominant symptoms in the later stages of pandemics.

In contrast to the downward trend on average level, there is a substantial proportion (at least 10 %) of individuals who showed an increased amount of symptoms of (un‐)specific anxiety and depression from T1 to T2 and to T4. Regarding the PHQ‐4 and the PHQ‐2, targeting symptoms of depression, at least 25 % of the sample showed an increase of symptom severity.

Moreover, it seems to be important to differentiate between different aspects of fears related to the pandemic. In contrast to general and economic fears, for example, the fear of the social consequences first increased from the beginning of the pandemic to the next assessment four weeks later. This may be due to the ongoing strict social distancing measures. In parallel to the stepwise easing of the restrictions of social contact at the third and fourth assessments, also the level of social worries decreased.

Besides the progression of symptoms, our study provides relevant results regarding protective and risk factors in dealing with the COVID‐19 pandemic. These results build an empirical basis for existing recommendations (IASC, 2020; IFRC, 2020; WHO, 2020). All examined protective and risk factors showed significant associations with (at least one but mostly several or even all) variables of anxiety and psychological distress four and ten weeks after the baseline relative to the baseline values. Self‐efficacy, normalization, maintaining social contacts, and knowledge where to get medical support were associated with fewer symptoms relative to baseline. Suppression, reduced healthy diet, reduced physical activity, more substance abuse, and a longer daily average time of thinking about the pandemic were longitudinally associated with higher levels of psychological strain.

While some of the factors showed stable associations with all outcomes (e.g., substance abuse), others did not. For example, physical activity is recommended for the prevention and coping with mental health problems (Arora & Grey, 2020; Diamond & Waite, 2020) and was associated with a better mental health in the pandemic (Petzold, Bendau, Plag, Pyrkosch, Mascarell Maricic, et al., 2020; Pieh et al., 2020; Stanton et al., 2020), but on a longitudinal perspective, it showed only a predictive value regarding the subjective evaluation of one’s anxiety as exaggerated and generating burdens in daily life. Moreover, for example, health‐related self‐efficacy showed stronger associations on the long than on the short run. Those pattern of results give a hint that the results distinguish between different outcome variables—for example, with respect to instruments and time perspectives. This methodological matter should be concerned in future studies.

It can be assumed that the application of maladaptive coping strategies (e.g., consuming alcohol, tobacco, and/or unhealthy food) and the severity of symptoms of anxiety and distress increase reciprocally (Bommele et al., 2020; Kim et al., 2020; Sidor & Rzymski, 2020). Maladaptive strategies may increase symptom burden which in turn may result in a stronger application of unhealthy coping behavior. Nevertheless, it should be remarked that a substantial proportion of people suffering from high levels of distress in the context of the pandemic reduced smoking (Bommele et al., 2020), respectively, drinking (Kim et al., 2020).

We examined only risk and protective factors that are probably valid for the general population and can be modified to prevent or reduce symptom burden: respectively, by the enhancement of protective variables and the counterbalancing/reduction of risk factors. Therefore, those factors should be targeted on individual and on a broader societal level (Vinkers et al., 2020). This requires particularly clear communication and psychoeducation. In addition, special attention should be paid to potentially more vulnerable groups.

Strengths of this study are the early start of recruitment with baseline data from a situation with heavily increasing case numbers in Germany. Furthermore, we did not only measure general psychopathology like anxiety and depression but also included a validated measure of specific anxiety regarding COVID‐19 and potential risk and protective factors. However, our study cannot entirely avoid some limitations. To keep the survey short, we used the PHQ‐4 as a brief assessment of the umbrella term psychological distress, respectively, the screening subscales for anxiety and depression. Although the construct validity as well as the sensitivity in general population samples has been shown to be moderate to good (Löwe et al., 2010), the usage of the PHQ‐4 does not substitute a detailed survey of depression and anxiety and should be rather interpreted as a rough approximation. The recruitment of the study sample as a convenience sample of the general population was mainly done through social media. This might have led to sample bias because individuals who frequently use social media or were especially interested or affected by the topic may have been more likely to participate. This might be a factor that the majority of our sample is rather young. Besides a lower average age, also regarding the higher percentage of participants working in a medical context, the higher average level of education and the higher gender imbalance our sample differs from the demographics in the general population in Germany (Bundesinstitut für Bevölkerungsforschung, 2020). Those limitations reduce the generalizability of our results and should be considered when interpreting the results as well that the sample is limited to the population in Germany. Furthermore, because of the lack of data prior to the pandemic, our study is not able to draw conclusions regarding the change of symptoms relative to their state before the pandemic and cannot differentiate clearly between pre‐existing symptoms and those who occurred new in the context of the pandemic. In contrast, our survey is able to describe the progress of symptoms during the ongoing pandemic which provides other—but also meaningful—information. In this context, it is important to consider the rather descriptive, observational, and explorative nature of this article which lays a broad basis for further research but cannot differentiate clearly the impact of single factors associated with the severity of symptoms in detail. For multiple testing was only corrected in the analysis of the changes in the outcome variables over time but not in the analysis of risk and protective factors. We decided to present the results in this way to give researchers a direct impression of the correlations of a large number of variables and points of measurement to lay the basis for the formulation of more specific hypotheses and more elaborated analysis in future studies.

Summing up, our findings provide important insight into the longitudinal changes of symptoms of psychological distress along the first three months of the COVID‐19 pandemic in Germany. Furthermore, we were able to reaffirm the anticipated protective and risk factors that were extracted from previous cross‐sectional studies (Bäuerle et al., 2020; Fullana et al., 2020; González‐Sanguino et al., 2020; Petzold, Bendau, Plag, Pyrkosch, Mascarell Maricic, et al., 2020) and recommendations of the WHO (2020) and other institutions (IASC, 2020; IFRC, 2020). Those factors should be targeted in future research as well as in preventive and therapeutic interventions to buffer the potential negative impact of the COVID‐19 pandemic on mental health and may be useful in dealing with potential other future crises, too.

## CONFLICT OF INTEREST

The authors declare that there is no conflict of interest.

## ACKNOWLEDGEMENT

Open access funding enabled and organized by Projekt DEAL.

## AUTHOR CONTRIBUTION

**Antonia Bendau involved in** literature research, conceptualization of the study, questionnaire construction, data collection, data preparation, data analysis, data interpretation, and writing. **Jens Plag involved in** literature research, conceptualization of the study, ethics committee communication, questionnaire construction, data collection, and critical review. **Stefanie Kunas involved in** literature research, data preparation, and critical review. **Sarah Wyka involved in** literature research and critical review. **Andreas Ströhle involved in** primary conceptualization of the study, ethics committee communication, questionnaire construction, data interpretation, and critical review. **Moritz Bruno Petzold involved in** literature research, conceptualization of the study, questionnaire construction, data collection, data interpretation, and writing.

## ETHICAL APPROVAL

The authors assert that all procedures contributing to this work comply with the ethical standards of the relevant national and institutional committees on human experimentation and with the Helsinki Declaration of 1975, as revised in 2008.

### Peer Review

The peer review history for this article is available at https://publons.com/publon/10.1002/brb3.1964.

## Supporting information

Supplementary Material

## Data Availability

Data are available from the corresponding author on reasonable request.
