# Supplementary material for: Longitudinal changes in anxiety and psychological distress, and associated risk and protective factors during the first three months of the COVID‐19 pandemic in Germany
Source: Brain Behav. 2020 Nov 23;11(2):e01964. doi: 10.1002/brb3.1964 (PMC7744907; doi:10.1002/brb3.1964)
Supplement: Supplementary file 1 — Supplementary Material [file BRB3-11-e01964-s001.docx]

**Supplement**

**Longitudinal changes in anxiety and psychological distress, and associated risk and protective factors during the first three months of the COVID-19 pandemic in Germany**

**Figure S1. Flow diagram of the participation in the four assessment waves (T1, T2, T3, and T4)**

Analysed T2 (*N* = 1819)

Analysed T4 (*N* = 1349)

Participated at T1 and gave consent to be contacted for follow-ups (*N* = 2963)

Excluded (*N* = 1108)

• Did not participate in at least one
 of the follow-ups (T2, T3 or T4).

Analysed T1 (*N* = 1855)

*N* = 1336

Included new participants at T2
 (*N* = 483)

• Did not already participate at T1

• Did participate in at least one of
 the follow-ups (T3 or T4)

*N* = 940

Analysed T3 (*N* = 1538)

Total sample: *N* = 2376
completed at least two waves

• *N* = 1070 completed two waves

• *N* = 803 completed three waves

• *N* = 503 completed four waves

*N* = 964

**Table S1 – Demographics of the subsamples of the four different measurement waves (T1, T2, T3, and T4)**

| **Point of measurement** | **T1 (*N* = 1855)** | | **T2 (*N* = 1819)** | | **T3 (*N* = 1538)** | | **T4 (*N* = 1349)** | |
| --- | --- | --- | --- | --- | --- | --- | --- | --- |
| **Characteristic** | **Mean (SD); Range** | | **Mean (SD); Range** | | **Mean (SD); Range** | | **Mean (SD); Range** | |
| **Age (years)** | 38.3 (12.0); 18-82 | | 38.6 (12.2); 18 - 76 | | 39.9 (12.2); 18-77 | | 40.5 (12.4); 18-77 | |
| **Characteristic** | **N** | **%** | **N** | **%** | **N** | **%** | **N** | **%** |
| **Gender**  - Female  - Male  - Diverse | 1418  425  12 | 76.4  22.9  0.6 | 1408  402  9 | 77.4  22.1  0.5 | 1189  343  6 | 77.3  22.3  0.4 | 1050  288  11 | 77.8  21.3  0.8 |
| **Education level**  - University degree  - Higher education entrance qualification  - Secondary degree  - Lower secondary degree  - No school degree | 1027  582  215  25  6 | 55.4  31.4  11.6  1.3  0.3 | 1076  530  185  15  3 | 59.2  29.1  10.2  0.8  0.2 | 926  432  160  15  5 | 60.2  28.1  10.4  1.0  0.3 | 811  378  144  12  4 | 60.1  28.0  10.7  0.9  0.3 |
| **Having children**  - Yes  - No | 746  1109 | 40.2  59.8 | 720  1089 | 39.6  59.9 | 657  881 | 42.7  57.3 | 570  779 | 42.3  57.7 |
| **Relationship status**  - Single, widowed, divorced  - Solid relationship  - Married  - Other | 659  557  620  19 | 35.5  30.0  33.4  1.0 | 633  544  619  13 | 34.8  29.09  34.0  0.7 | 543  443  547  5 | 35.3  28.8  25.6  0.3 | 489  370  482  8 | 36.2  27.4  35.7  0.6 |
| **Working in medical context**  - Yes  - No | 320  1535 | 17.3  82.7 | 310  1483 | 17.0  81.5 | 234  1221 | 15.2  89.2 | 211  1042 | 15.6  77.2 |
| **Physical disease**  - Yes  - No | 228  1627 | 12.3  87.7 | 206  1578 | 11.3  87.2 | 172  1283 | 11.2  83.4 | 168  1115 | 12.5  82.7 |

**Table S2. Changes in different aspects of COVID-19 related fears**

| **My anxiety concerning Corona …** | | **… is exaggerated** | **… leads to limitations of my daily life** | **… leads to worse limitations of my daily life than the virus itself** |
| --- | --- | --- | --- | --- |
| **T1** (*N* = 1855) | *M* ± *SD* Median | 2.37 ± 1.24 2.00 | 2.44 ± 1.54 2.00 | 2.28 ± 1.44 2.00 |
| **T2** (*N* = 1808) | *M* ± *SD* Median | 2.18 ± 1.18 2.00 | 2.20 ± 1.44 2.00 | 2.11 ± 1.40 2.00 |
| **T3** (*N* = 1537) | *M* ± *SD* Median | 2.16 ± 1.18 2.00 | 2.10 ± 1.38 2.00 | 2.10 ± 1.40 2.00 |
| **T4** (*N* = 1345) | *M* ± *SD* Median | 2.06 ± 1.13 2.00 | 1.95 ± 1.29 1.00 | 1.93 ± 1.26 1.00 |
| **Difference**  **T2 – T1** (*N* = 1340) | *M* ± *SD* Median  10. Percentile  25. Percentile  75. Percentile  90. Percentile | -0.16 ± 1.20 0.00  -2.00  -1.00  0.00  1.00 | -0.21 ±  1.31 0.00  -2.00  -1.00  0.00  1.00 | -0.16 ± 1.43 0.00  -2.00  -1.00  0.00  1.00 |
| **Difference**  **T4 – T1** (*N* = 975) | *M* ± *SD* Median  10. Percentile  25. Percentile  75. Percentile  90. Percentile | -0.35 ± 1.23 0.00  -2.00  -1.00  0.00  1.00 | -0.50 ± 1.35 0.00  -2.00  -1.00  0.00  1.00 | -0.39 ± 1.34 0.00  -2.00  -1.00  0.00  1.00 |

**Table S3. Associations of different aspects of COVID-19 related fears (at T2 and T4) with protective and risk factors (at T1). Pearson’s partial correlations with partialization of the T1-values of the respective fear-variable.**

| **My anxiety concerning Corona …** | **… is exaggerated** | **… leads to limitations of**  **my daily life** | **… leads to worse limitations of my daily life than the virus itself** |
| --- | --- | --- | --- |
| **Protective factors [T1]** |  |  |  |
| Self-efficacy general | **r = -.15 (p < .001***) r = -.17 (p < .001***)** | **r = -.12 (p < .001***) r = -.20 (p < .001***)** | **r = -.18 (p < .001***) r = -.19 (p < .001***)** |
| Self-efficacy health | **r = -.13 (p < .001***) r = -.19 (p < .001***)** | **r = -.11 (p < .001***) r = -.20 (p < .001***)** | **r = -.10 (p < .001***) r = -.18 (p < .001***)** |
| Self-efficacy social | **r = -.11 (p < .001***)** r = -.09 (p = .005**) | r = -.08(p = .003**) **r = -.13 (p < .001***)** | **r = -.15 (p < .001***) r = -.15 (p < .001***)** |
| Self-efficacy economic | r = -.05 (p = .067) r = -.00 (p = .893) | r = -.07 (p = .012*) r = -.03 (p = .418) | **r = -.10 (p < .001***)** r = -.07 (p = .023*) |
| Normalization | **r = -.21 (p < .001***) r = -.18 (p < .001***)** | **r = -.16 (p < .001***) r = -.14 (p < .001***)** | **r = -.16 (p < .001***) r = -.14 (p < .001***)** |
| Social Contacts | **r = -.10 (p < .001***) r = -.11 (p = .001***)** | r = -.05 (p = .066) **r = -.12 (p < .001***)** | r = -.07 (p = .014*) **r = -.11 (p < .001***)** |
| Medical Support | r = -.08 (p = .005**) **r = -.11 (p = .001***)** | r = -.07 (p = .011*) **r = -.11 (p < .001***)** | r = .02 (p = .533) r = -.05 (p = .097) |
| Psych. Support | r = -.03 (p = .210) r = -.08 (p = .014*) | r = -.02 (p = .401) r = -.08 (p = .017*) | r = .02 (p = .592) r = -.04 (p = .249) |
| **Risk factors [T1]** |  |  |  |
| Suppression | **r = .14 (p < .001***) r = .20 (p < .001***)** | **r = .13 (p <.001***) r = .13 (p <.001***)** | **r = .14 (p <.001***) r = .18 (p <.001***)** |
| Reduced physical activity | r = .06 (p = .035*) r = .09 (p = .007**) | r = .00 (p = .896) r = .08 (p = .012*) | r = .06 (p = .039*) **r = .10 (p = .001***)** |
| Reduced healthy diet | r = .02 (p = .505 **r = .10 (p = .001***)** | r = .06 (p = .026*) **r = .13 (p = .002**)** | r = .08 (p = .003**) **r = .10 (p = .002**)** |
| More substance use | r = .04 (p = .117) r = .09 (p = .004**) | r = .08 (p = .005**) **r = .10 (p = .001***)** | r = .09 (p = .001***) **r = .14 (p = .001***)** |
| h/day thinking about C. | **r = .10 (p < .001***) r = .10 (p = .001***)** | **r = .10 (p = .001***) r = .10 (p = .003**)** | **r = .10 (p < .001***)** r = .06 (p = .049*) |

*significant at .05-level , **sig..01-level ***sig. at .001-level; bold values represent significant values of a size of at least .1

**Table S4. Associations of different aspects of COVID-19 related fears (at T2 and T4) with protective and risk factors (at T1). Pearson’s partial correlations with partialization of the T1-values of the respective fear-variable.**

| **I am afraid…** | **… to get infected**  **with Corona** | **… of the conse-quences of the Corona Pandemic on my life** | **… of the consequences**  **for my health if I get infected** | **… of the social**  **consequences**  **of Corona** | **… of the economic**  **consequences of Corona on my life** |
| --- | --- | --- | --- | --- | --- |
| **Protective factors [T1]** |  |  |  |  |  |
| Self-efficacy general | r = -.09 (p = .001^***^) **r = -.10 (p = .002^**^)** | **r = -.15 (p < .001^***^) r = -.15 (p < .001^***^)** | r = -.05 (p = .084) r = -.09 (p = .008^**^) | **r = -.12 (p < .001^***^)** r = -.09 (p = .007^**^) | **r = -.10 (p < .001^***^) r = -.13 (p < .001^***^)** |
| Self-efficacy health | **r = -.19 (p < .001^***^) r = -.24 (p < .001^***^)** | r = -.08 (p = .011^*^) **r = -.13 (p < .001^***^)** | **r = -.19 (p < .001^***^) r = -.23 (p < .001^***^)** | r = -.01 (p = .690) r = -.09 (p = .004^**^) | r = -.06 (p = .026^*^) r = -.09 (p = .004^**^) |
| Self-efficacy social | r = .04 (p = .154) r = .00 (p = .922) | **r = -.19 (p < .001^***^) r = -.16 (p < .001^***^)** | r = .03 (p = .221) r = .01 (p = .879) | **r = -.18 (p < .001^***^) r = -.22 (p < .001^***^)** | **r = -.10 (p < .001^***^) r = -.15 (p < .001^***^)** |
| Self-efficacy econom. | r = -.03 (p = .332) r = -.02 (p = .575) | **r = -.12 (p < .001^***^) r = -.19 (p < .001^**^)** | r = -.03 (p = .305) r = .00 (p = .891) | r = -.08 (p = .002**) **r = -.11 (p = .001^***^)** | **r = -.23 (p < .001^***^) r = -.35 (p < .001^***^)** |
| Normalization | r = -.02 (p = .576) r = -.04 (p = .207) | r = -.06 (p = .032^*^) r = -.06 (p = .055) | r = .01 (p = .799) r = -.01 (p = .686) | r = -.08 (p = .003^**^) **r = -.11 (p = .001^***^)** | r = -.09 (p = .008^**^) r = -.05 (p = .129) |
| Social Contacts | r = .05 (p = .078) r = .03 (p = .345) | r = -.09 (p = .001^***^) r = -.04 (p = .182) | r = .02 (p = .447) r = -.00 (p = .975) | r = -.08 (p = .003^**^) r = -.03 (p = .406) | r = -.09 (p = .001^***^) r = -.05 (p = .132) |
| Medical Support | r = -.00 (p = .972) r = -02 (p = .481) | r = -.05 (p = .094) **r = -.10 (p = .003^**^)** | r = -.01 (p = .137) r = .01 (p = .784) | r = -.01 (p = .839) r = -.04 (p = .173) | r = -.08 (p = .006^**^) **r = -.13 (p < .001^***^)** |
| Psych. Support | r = .03 (p = .308) r = .05 (p = .151) | r = -.04 (p = .134) r = -.01 (p = .826) | r = -.01 (p = .668) r = -.01 (p = .818) | r = -.03 (p = .260) r = .02 (p = .592) | r = -.02 (p = .555) r = -.03 (p = .446) |
| **Risk factors [T2]** |  |  |  |  |  |
| Suppression | r = .02 (p = .571) r = .07 (p = .033^*^) | **r = .11 (p < .001^***^) r = .14 (p < .001^***^)** | r = .02 (p = .430) **r = .12 (p < .001^***^)** | r = .07 (p = .007^**^) r = .08 (p = .017^*^) | r = .09 (p = .001^***^) r = .05 (p = .112) |
| Reduced phys. activity | r = .02 (p = .446) r = -.01 (p = .878) | r = .04 (p = .193) r = .05 (p = .153) | r = .03 (p = .244) r = .04 (p = .244) | r = .04 (p = .207) r = .03 (p = .365) | r = .04 (p = .183) r = .03 (p = .384) |
| Reduced healthy diet | r = -.04 (p = .165) r = -.01 (p = . 780) | r = .06 (p = .019^*^) r = .09 (p = .006^**^) | r = -.06 (p = .023^*^) r = .02(p = .526) | r = .04 (p = .125) r = .05 (p = .103) | r = .08 (p = .006^**^) r = .04 (p = .225) |
| More substance use | r = -.05 (p = .073) r = -.01 (p = .658) | r = .09 (p = .001^***^) r = .04 (p = .233) | r = -.02 (p = .405) r = .02 (p = .583) | **r = .10 (p < .001^***^)** r = .07 (p = .040^*^) | r = .05 (p = .066) r = -.00 (p = .974) |
| h/day thinking about | r = .05 (p = .065) r = .00 (p = .993) | r = .05 (p = .076) **r = .10 (p = .003^**^)** | r = .04 (p = .175) r = .01 (p = .762) | **r = .10 (p = .001^***^)** r = .04 (p = .272) | r = .02 (p = .563) r = .07 (p = .036^*^) |

*significant at .05-level, **sig. at .01-level ***sig. at .001-level; bold values represent significant values of a size of at least .1
